# Could persistent extravascular fibrin deposition link microvascular and interstitial pathology in long COVID?

**DOI:** 10.3389/fmed.2026.1964697

**Published:** 2026-09-23

**Authors:** Keiko Kubo

**Affiliations:** HSG Interstitial Research Lab (HIRL), Chiba, Japan

**Keywords:** endothelial dysfunction, extravascular coagulation, fibrin deposition, immunothrombosis, impaired fibrinolysis, interstitial pathology, long COVID, post-acute sequelae of SARS-CoV-2 infection (PASC)

## Abstract

Long COVID is associated with persistent endothelial dysfunction, microvascular impairment, coagulation abnormalities, impaired fibrinolysis, and tissue hypoxia. Although these intravascular abnormalities are increasingly documented, less attention has been paid to whether vascular injury may also generate persistent pathology in the extravascular interstitial compartment. We propose chronic intra- and extravascular coagulation (CIEC) as a continuous pathological mechanism in which COVID-associated immunothrombosis and endothelial injury may be followed by vascular leakage, local extravascular fibrin formation, and incomplete resolution of a fibrin-containing provisional matrix. At a different level, the dual-axis microvascular-interstitial model (DMIM) describes how abnormalities on both sides of the vascular wall may interact functionally: impaired microvascular tissue supply (Axis 1) together with abnormal interstitial mechanics and diffusion (Axis 2) may contribute to tissue dysfunction and symptoms. This framework links established microvascular pathology with a proposed extravascular component and provides testable predictions for histological, biochemical, molecular-imaging, microcirculatory, and biomechanical studies in long COVID.

## 1 Introduction

Long COVID is increasingly recognized as a heterogeneous multisystem condition in which vascular, coagulation, immunological, autonomic, and metabolic abnormalities may interact to produce persistent symptoms (1, 2). Reported vascular and coagulation-related findings include persistent endothelial dysfunction and microvascular impairment (3–7), fibrin amyloid microclots with impaired fibrinolysis (8–10), platelet and complement abnormalities, and chronic immune activation driven in part by persistent viral antigens (11–14). Quantitative studies have also reported post-COVID abnormalities in skin microcirculation, retinal and conjunctival microvasculature, and cerebral blood flow (15–18). These findings indicate a substantial intravascular component of post-COVID pathophysiology, although long COVID is clinically diverse and unlikely to be explained by any single pathway (19, 20).

The present hypothesis builds partly on the author's previously published, qualitative clinical observations underlying the Hematic Stiff Gelsis (HSG) concept (21). These observations arose during manual treatment and were not prospectively collected experimental data. HSG denotes a putative viscoelastic material inferred from subjective palpatory findings; its biological composition remains unknown. As described in the earlier report, symptomatic changes following intervention were primarily clinically reported rather than evaluated using standardized objective outcome measures. The palpated substrate was not identified using histological, biochemical, imaging, or biomechanical measurements, and the findings have not been independently reproduced under blinded conditions. These observations provide a rationale for hypothesis development but do not establish the existence of a specific material, treatment efficacy, or causality. The present article discusses these previously published observations without presenting new patient-level data or reanalysing clinical records.

We integrate these observations with published evidence on endothelial injury, microvascular dysfunction, coagulation abnormalities, impaired fibrinolysis, vascular permeability, and extracellular-matrix remodeling, and propose chronic intra- and extravascular coagulation (CIEC) as a mechanistic continuum extending from COVID-associated immunothrombosis and endothelial injury within the vasculature to vascular leakage and a possible blood-derived fibrin-containing substrate within the interstitium. CIEC therefore describes a proposed sequence of pathological events across the vascular boundary, rather than a functional symptom model. The downstream functional consequences of simultaneous intravascular and interstitial abnormalities are considered separately in the dual-axis microvascular-interstitial model (DMIM).

## 2 Extravascular fibrin formation, stabilization, and resolution

Microvascular permeability determines whether plasma proteins reach the extravascular compartment. Experimental work has shown that increased permeability can permit fibrinogen and other coagulation proteins to leave the vascular lumen and enter inflamed or injured tissue (22–24). However, fibrinogen extravasation alone does not establish fibrin formation. Converting fibrinogen to fibrin requires local coagulation activity, including tissue factor-dependent generation of factor Xa and thrombin. Thrombin cleaves fibrinogen to fibrin and activates factor XIII (FXIII); activated FXIII cross-links fibrin and can increase mechanical stability and resistance to fibrinolysis (25). The extent to which platelets or other blood-derived cells contribute to this process in chronically inflamed extravascular tissue is context-dependent and has not been established in long COVID.

Extravascular fibrin formation therefore depends on a local balance rather than following inevitably from vascular leakage. Tissue factor-driven coagulation is opposed by endogenous anticoagulant mechanisms, including tissue factor pathway inhibitor, antithrombin, and the thrombomodulin-protein C system, as well as by fibrinolysis. In wounds and tumors, fibrin can form a provisional matrix that retains water and solutes, supports cell migration and angiogenesis, and is subsequently degraded, cleared, or replaced during extracellular-matrix remodeling (23, 24, 26, 27). Most of the foundational mechanistic evidence cited here derives from *in vitro* studies, animal models, and human pathological settings other than long COVID. There are currently no direct human data demonstrating a chronic, widespread fibrin-containing interstitial matrix in long COVID.

Resolution is also an active biological process. Extravascular fibrin can undergo plasmin-dependent degradation and macrophage-mediated endocytic clearance; *in vivo* work in mice has identified CCR2-positive macrophages as important mediators of extravascular fibrin removal (28). Lymphatic vessels normally remove excess interstitial fluid and transport macromolecules from tissues (29). Persistent extravascular material could therefore reflect repeated leakage and formation, impaired fibrinolysis, insufficient cellular or lymphatic clearance, abnormal matrix remodeling, or a combination of these processes. We use the term fibrin-containing provisional matrix for the proposed biological substrate; viscoelasticity refers only to its hypothesized mechanical property and not to a proven chemical composition.

We hypothesize that, in a subset of patients with long COVID, repeated or persistent vascular leakage together with incomplete resolution could allow a fibrin-containing provisional matrix to persist or undergo abnormal remodeling. It has not been established whether such a matrix corresponds to HSG (the putative viscoelastic material perceived manually in affected tissue regions), to gel-like blood-containing material occasionally observed during medical cupping, or to neither. Compositional equivalence among these phenomena and their identity with fibrin-containing material require direct biochemical and histological validation.

## 3 Relationship to immunothrombosis, COVID-19-associated coagulopathy, and DIC

Acute COVID-19 is characterized by endothelial injury, immunothrombosis, and COVID-19-associated coagulopathy (CAC), with microvascular thrombosis occurring particularly in severe disease. CAC and disseminated intravascular coagulation (DIC) are distinct clinical and laboratory entities, but they share thromboinflammatory mechanisms, including endothelial injury, immunothrombosis, activation of coagulation, dysregulation of fibrinolysis, and microcirculatory impairment (30–35). CAC differs from overt DIC in its typical laboratory phenotype and does not necessarily fulfill diagnostic criteria for consumptive coagulopathy. The intravascular component of CIEC belongs to this established spectrum of COVID-associated endothelial injury and immunothrombosis. What the present framework adds is the possibility that vascular leakage and local coagulation may generate persistent extravascular fibrin-containing pathology.

These shared mechanisms do not imply diagnostic equivalence. Localized immunothrombosis is an intravascular process, whereas inflammatory extravascular fibrin formation occurs outside the vascular lumen; evidence for the former does not by itself establish the latter. CIEC is proposed as a possible persistent or recurrent process linking these compartments, but it has no established laboratory phenotype or validated diagnostic criteria, and its chronic extravascular component remains unproven in long COVID. Long COVID is not presented here as chronic or compensated DIC.

## 4 The CIEC hypothesis: a proposed pathological process

We define CIEC as a hypothesized continuous pathological mechanism that begins with, or develops from, intravascular immunothrombosis and endothelial injury. It then extends across the vascular wall through leakage of fibrinogen and other plasma-derived proteins into the interstitium. If local coagulation occurs and resolution is incomplete, a fibrin-containing provisional matrix may persist or undergo abnormal remodeling outside the vascular lumen. CIEC therefore describes the proposed mechanistic sequence from intravascular immunothrombotic pathology to extravascular interstitial pathology, rather than the symptoms produced by these abnormalities.

In CIEC, “chronic” refers primarily to persistence or recurrence of this pathological process beyond the acute phase. It does not necessarily imply continuous active coagulation at all times. The term may also encompass incomplete resolution and persistence of pathological products or downstream tissue abnormalities, including intravascular microthrombotic changes and a hypothesized extravascular fibrin-containing interstitial substrate.

The proposed temporal sequence is conditional rather than deterministic: persistent or recurrent triggers may promote endothelial dysfunction and leakage; microvascular abnormalities may reduce tissue supply; and, in parallel, extravasated coagulation substrates may undergo local fibrin formation. Normal fibrinolysis, macrophage-mediated clearance, lymphatic clearance, and matrix remodeling would favor resolution. CIEC would require that formation or recurrence outpace these resolution processes. Failed resolution, rather than coagulation alone, is therefore central to the hypothesized chronic extravascular component.

Why only some individuals develop persistent symptoms after SARS-CoV-2 infection remains unresolved and is beyond the primary scope of this article. Nevertheless, several factors would plausibly influence whether the proposed process persists: the magnitude or recurrence of endothelial injury, ongoing inflammatory or antigenic triggers, fibrinolytic capacity, macrophage and lymphatic clearance, and extracellular-matrix remodeling. These factors are presented as testable modifiers rather than established determinants of long-COVID susceptibility (Figure 1).

**Figure 1:**
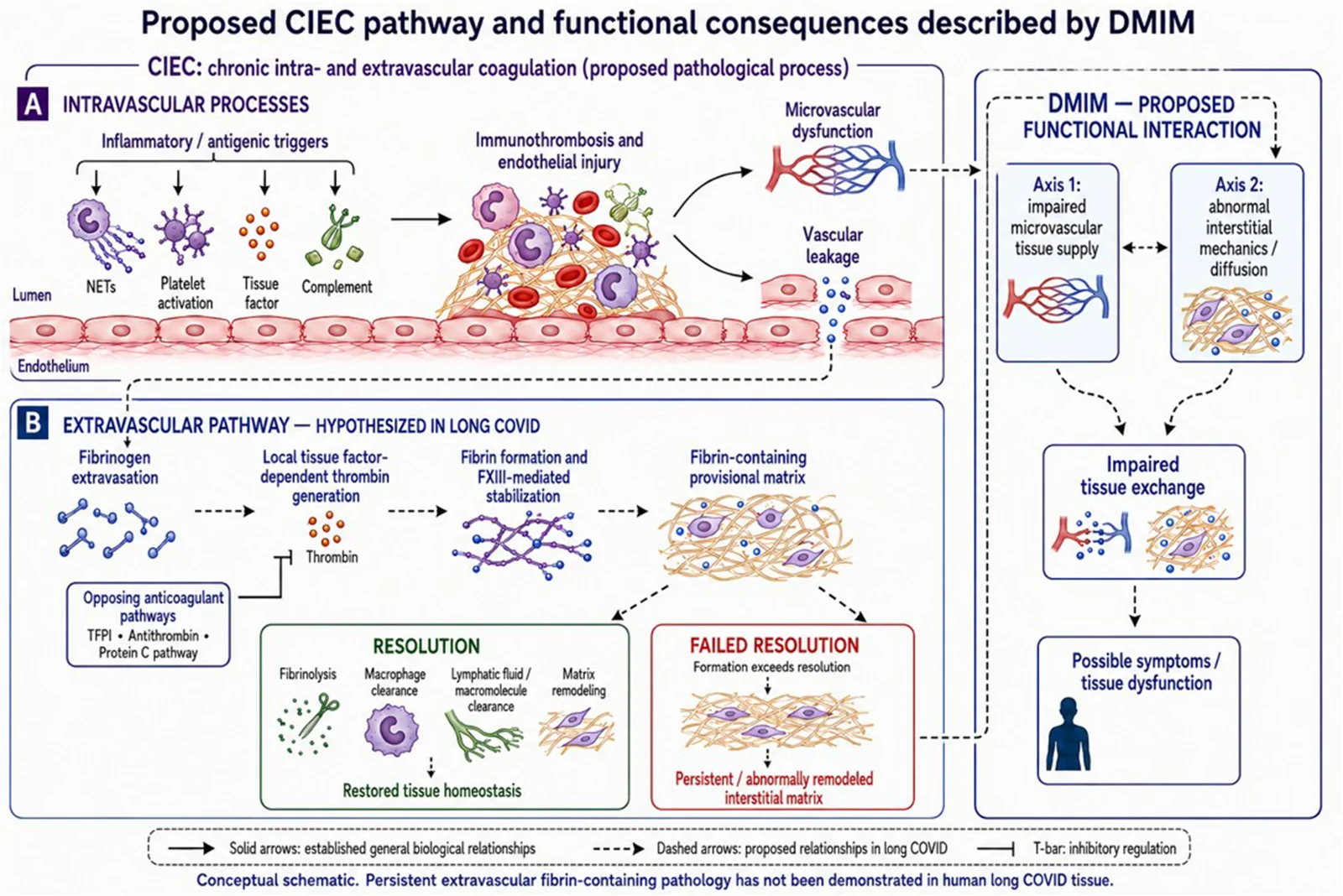
Proposed CIEC pathway from immunothrombosis to interstitial pathology and the functional consequences described by DMIM. The schematic illustrates the proposed relationship between chronic intra- and extravascular coagulation (CIEC) and the dual-axis microvascular-interstitial model (DMIM). CIEC describes a hypothesized pathological continuum beginning with intravascular immunothrombosis and endothelial injury. Persistent or recurrent endothelial dysfunction may contribute to microvascular impairment and vascular leakage, allowing fibrinogen and other plasma-derived proteins to enter the interstitium. Where local thrombin generation occurs, extravasated fibrinogen may be converted to fibrin, forming a fibrin-containing provisional matrix. Normal fibrinolysis, macrophage-mediated clearance, lymphatic clearance, and matrix remodeling would favor resolution, whereas incomplete resolution or recurrent formation is hypothesized to promote persistence or abnormal remodeling of the interstitial substrate. In CIEC, “chronic” refers to persistence or recurrence of the pathological process beyond the acute phase. The term may also encompass incomplete resolution and persistence of its products or downstream tissue abnormalities; it does not necessarily imply continuous active coagulation at all times. DMIM conceptualizes the resulting abnormalities functionally: Axis 1 represents impaired microvascular tissue supply, whereas Axis 2 represents abnormal interstitial mechanics and diffusion. Interaction between these two axes is hypothesized to contribute to tissue dysfunction and symptoms. The figure distinguishes established or previously reported processes from hypothetical transitions. The proposed chronic extravascular fibrin-containing interstitial substrate has not been directly demonstrated in human long COVID tissue.

Whether persistence requires an ongoing inflammatory or antigenic trigger, or whether local feedback between vascular dysfunction and interstitial remodeling could sustain the process after the initiating trigger has subsided, remains unresolved. These alternatives could be distinguished in longitudinal studies assessing candidate triggers alongside vascular and interstitial abnormalities. Persistence after a trigger becomes undetectable would not, by itself, prove a self-sustaining mechanism.

## 5 Discussion

### 5.1 The dual-axis microvascular-interstitial model (DMIM)

CIEC and DMIM represent complementary but distinct levels of the proposed framework. CIEC is a mechanistic hypothesis describing how intravascular immunothrombotic pathology may extend continuously into the extravascular interstitium through endothelial injury, vascular leakage, local fibrin formation, and failed resolution. DMIM is a functional model of how abnormalities in the two compartments may produce tissue dysfunction and symptoms: Axis 1 represents impaired microvascular tissue supply, whereas Axis 2 represents abnormal interstitial mechanics and diffusion. In other words, CIEC concerns how the intra- and extravascular pathology may develop, whereas DMIM concerns how the resulting abnormalities may interact to impair tissue function. DMIM is therefore not synonymous with CIEC and could, in principle, arise from other pathological processes.

Axis 1 (microvascular/intravascular) is impaired functional tissue blood supply arising from established or previously proposed mechanisms such as microvascular dysfunction, microvascular loss, microthrombotic obstruction, and abnormal hemodynamics. Quantitative post-COVID studies have demonstrated reduced microvascular flow or density in several tissues (15–18), and the blood-supply reduction (SR) mechanism proposed by Koutsiaris integrates these findings as a potential explanation for reduced peripheral mass transport and oxygen delivery (36, 37). The present hypothesis does not claim novelty for Axis 1.

Axis 2 (interstitial/extravascular) is the proposed accumulation or abnormal remodeling of a fibrin-containing provisional matrix, with increased viscoelasticity, raised tissue pressure or mechanical restriction, and increased resistance to diffusion. Axis 2 remains hypothetical in long COVID because its substrate has not been directly demonstrated in affected human tissues.

When both axes coexist, two sequential steps could limit tissue oxygen and nutrient delivery: reduced convective supply to the exchange microvasculature, followed by increased resistance across the extravascular/interstitial pathway. This dual limitation could contribute to a supply-demand mismatch during exertion, and to delayed recovery. Such a mechanism may be relevant to fatigue or post-exertional malaise in some patients, but it is not proposed as a universal explanation for the full spectrum of long-COVID symptoms.

### 5.2 Thromboinflammatory mediators, endothelial injury, and vascular leakage

Neutrophil extracellular traps (NETs), extracellular histones, complement activation, von Willebrand factor, platelet-leukocyte interactions, and inflammatory mediators are interconnected components of immunothrombosis and endothelial injury (35, 38–40). COVID-19 studies have demonstrated systemic microvascular endotheliopathy, glycocalyx injury, and post-COVID microvascular abnormalities (15, 41, 42). These findings support the established side of the proposed sequence: inflammatory and thrombotic processes can damage the endothelial barrier and alter microvascular function.

Barrier dysfunction may increase the extravasation of fibrinogen and other plasma proteins into inflamed tissue (23, 24, 35). This does not mean, however, that intravascular immunothrombi themselves physically extend into the interstitial compartment. The hypothesized bridge is instead indirect: an injured, permeable vascular environment provides the substrates and local conditions from which an independent extravascular fibrin-forming process could arise.

### 5.3 From vascular leakage to persistent interstitial lesions: relationship to HSG

Plaut proposed that inflammatory activation of fascial myofibroblasts and subsequent extracellular-matrix remodeling could contribute to widespread tissue stiffening, mechanical compression, impaired perfusion, and chronic symptoms in long COVID (43). This is a plausible downstream model of fascia-related biomechanical dysfunction. The present hypothesis raises a prior question: could plasma-derived material leaking from injured microvessels supply a provisional substrate that persists long enough to influence these remodeling processes?

In wounds and tumors, extravascular fibrin forms a provisional matrix that supports fibroblast migration, stromal organization, and angiogenesis before being degraded or replaced during remodeling (24, 26, 27). Extending this established biology to long COVID remains an inference, not an observation. We hypothesize that recurrent leakage combined with inadequate resolution by fibrinolytic, cellular, lymphatic, or matrix-remodeling mechanisms could leave a persistent or repeatedly renewed fibrin-containing interstitial substrate. Such material could precede, coexist with, or amplify later tissue stiffening.

HSG is the earlier clinically derived concept from which this extravascular component developed (21). It denotes a hypothesized viscoelastic material perceived manually within affected tissue regions and was originally proposed to involve blood-derived interstitial material. The current CIEC framework does not establish that HSG is fibrin, nor that HSG, material observed during medical cupping, and a fibrin-containing provisional matrix are the same substance. These proposed relationships require direct biochemical, histological, and biomechanical testing.

Manual intervention may transiently alter local tissue mechanics, interstitial fluid distribution, fascial sliding, tissue pressure, microvascular perfusion, or lymphatic flow. Whether clinical improvement observed after such intervention results from redistribution or clearance of a specific pathological substrate, including fibrin-containing material, is unknown. The clinical observations should therefore be used to select tissue sites and design experiments; they are not evidence that a particular substrate has been removed.

### 5.4 Integration with existing models and related chronic syndromes

Long COVID encompasses multiple clinical phenotypes. In a large retrospective cohort, nine core long-COVID features were identified, including respiratory, fatigue/malaise, pain, cognitive, gastrointestinal, and neuropsychiatric manifestations (20). Cardiovascular long COVID is likewise considered mechanistically heterogeneous (19). Accordingly, we propose the CIEC/DMIM framework as one explanation in a subset of patients, particularly where microvascular dysfunction, exercise intolerance, pain, swelling, tissue mechanical symptoms, or other features plausibly related to impaired tissue exchange coexist.

The microvascular component of DMIM overlaps with existing models rather than replacing them. Fibrinaloid microclot hypotheses emphasize abnormal fibrin structure and impaired fibrinolysis within circulating blood (8–10), whereas the SR mechanism emphasizes reduced functional blood supply to exchange tissues downstream of microvascular loss and hemodynamic changes (36). These mechanisms can be incorporated within Axis 1. What distinguishes DMIM is its proposal that an additional resistance may exist after blood reaches the microcirculation, within the extravascular diffusion and mechanical environment of the interstitium.

Long COVID and ME/CFS share fatigue, cognitive dysfunction, autonomic symptoms, exercise intolerance, and post-exertional malaise. Overlapping immune, metabolic, autonomic, and vascular abnormalities have also been reported (2, 44). Similar impairment of brachial artery flow-mediated dilation has been reported in long COVID and ME/CFS (45), and hypercoagulability, platelet hyperactivation, and fibrinaloid microclots have been described in ME/CFS (46). Recent integrative models of ME/CFS likewise emphasize interacting vascular, immune, metabolic, and tissue-level processes rather than a single lesion (47). These parallels do not imply identical pathogenesis, but they do justify investigating shared microvascular and tissue-exchange mechanisms.

A small proteomic study of individuals with persistent symptoms following COVID-19 vaccination reported heterogeneous amyloid microclot deposits and changes in coagulation-, acute-phase-, and immune-related proteins, while acknowledging that unrecognized SARS-CoV-2 infection could not be excluded (48). Extending the CIEC/DMIM framework beyond long COVID therefore remains speculative.

### 5.5 Testable predictions and future directions

The central extravascular prediction is that a subset of clinically affected tissues contains diffuse or poorly demarcated interstitial material enriched in plasma-derived proteins, particularly fibrin(ogen), with altered hydration and mechanical properties. Because the predicted abnormality concerns tissue composition and rheology rather than a discrete mass, routine CT, routine MRI, and B-mode ultrasonography might all be normal and cannot by themselves confirm or exclude the hypothesis.

Testing should combine complementary methods rather than rely on a single imaging modality. These include histological and immunohistochemical assessment of fibrin(ogen) and other plasma-derived proteins; biochemical or proteomic tissue sampling where feasible; fibrin-targeted molecular imaging; quantitative MRI techniques sensitive to water mobility or macromolecular composition; ultrasound or MR elastography for mechanical properties; and independent microcirculatory measurements to assess perfusion and tissue supply (49–51).

Assays should distinguish fibrin from extravasated fibrinogen and verify localization outside vessel lumina. Increased stiffness or a fibrin(ogen) signal alone would not establish the proposed substrate. Positivity criteria should be prespecified using assay validation and appropriate control distributions rather than an arbitrary numerical threshold.

Prospective studies should determine whether, compared with matched controls, clinically affected tissues show:

- Increased interstitial fibrin(ogen), plasma-derived proteins, or coagulation products on histological, biochemical, or molecular-imaging assessment.
- Altered water content, diffusion, stiffness, or viscoelasticity that co-localizes with symptoms or independently assessed tissue findings.
- Associations between interstitial abnormalities and endothelial permeability, microvascular perfusion, oxygen extraction, coagulation activation, fibrinolytic impairment, or lymphatic dysfunction.
- Longitudinal changes in objective interstitial measures that track symptom fluctuation, post-exertional malaise, or recovery.
- Concordant changes in objective tissue measurements and symptoms after exploratory interventions intended to alter microvascular perfusion, tissue mechanics, or interstitial fluid dynamics.

A rigorous test will require pre-specified tissue sites, appropriate control groups, blinded analysis of images or samples, reproducible biomechanical assessment, and, where feasible, histological or biochemical validation. Repeated failure to detect the predicted extravascular material or associated mechanical abnormalities with adequately sensitive methods would argue against or substantially narrow the hypothesis.

The proposed extravascular component would be supported by reproducible evidence of a fibrin-containing matrix outside vessel lumina in affected tissues, with greater abundance than in appropriately matched controls. Its proposed functional relevance would require additional evidence linking this finding to altered tissue mechanics or diffusion and to clinical manifestations. Detection of fibrin alone would not establish the complete CIEC pathway or a causal role in symptoms.

### 5.6 Novelty of this paper

Neither the microvascular axis nor the previously proposed HSG concept is introduced for the first time in this article. The principal novelty is twofold: the mechanistic integration of established intravascular pathology with a hypothesized blood-derived extravascular substrate, and the explicit temporal proposal that vascular injury and leakage, conditional local fibrin formation, and failed resolution could generate a persistent interstitial abnormality. The framework further proposes and defines two complementary concepts: CIEC as the pathological process linking intravascular immunothrombosis to extravascular interstitial pathology, and DMIM as the functional model describing how impaired microvascular supply and interstitial mechanical/diffusion abnormalities may interact to generate tissue dysfunction and symptoms.

### 5.7 Limitations

This article proposes a conceptual, hypothesis-generating framework. No direct experimental evidence demonstrates the co-occurrence of persistent intravascular and extravascular coagulation in human long COVID. Chronic and widespread extravascular fibrin deposition has not been established histologically, biochemically, or by molecular imaging in affected patients. The composition, hydration, mechanical properties, anatomical distribution, and prevalence of the proposed interstitial substrate are unknown, as is its causal relationship with symptoms.

The clinical observations underlying the HSG hypothesis are subjective, were made by the author alone, and have not been independently reproduced under blinded conditions. The putative viscoelastic material perceived manually has not been biologically identified, and symptomatic improvement following manual intervention does not establish mechanism or causality. The relationship between HSG, material observed during medical cupping, and a fibrin-containing provisional matrix remains speculative. Because the observations were made by the author who formulated the hypothesis, observer expectancy and confirmation bias cannot be excluded.

Several steps in the proposed sequence are extrapolated from wound healing, tumor stroma, inflammatory biology, and animal studies. In particular, no study has yet demonstrated the conditions required for persistent local thrombin generation, FXIII-mediated stabilization, failed fibrinolysis, impaired macrophage or lymphatic clearance, and abnormal matrix remodeling in long COVID. Finally, long COVID is heterogeneous; the CIEC/DMIM framework is not proposed as a universal explanation and may be relevant only to selected phenotypes or disease stages.

## 6 Conclusion

Persistent vascular and microcirculatory abnormalities are established components of long COVID, whereas chronic extravascular fibrin-containing pathology remains unproven. CIEC proposes a continuous mechanism by which intravascular immunothrombosis and endothelial injury may progress across the vascular boundary through leakage, local fibrin formation, and failed resolution to produce persistent interstitial pathology in a subset of patients. In CIEC, chronicity may reflect persistence or recurrence of the pathological process or incomplete resolution with persistence of its products or downstream tissue abnormalities, rather than continuous active coagulation at all times. DMIM operates at a different level: it describes how the resulting intravascular impairment of tissue supply and extravascular abnormalities of tissue mechanics and diffusion may interact to generate dysfunction and symptoms. This framework does not replace existing microclot, microvascular, immune, or metabolic models. Its value lies in generating direct, falsifiable questions about the extravascular compartment. Histological, biochemical, molecular-imaging, microcirculatory, and biomechanical studies are required to determine whether the proposed extravascular substrate exists in long COVID and, if so, whether it contributes meaningfully to symptoms.

## Data Availability

The original contributions presented in the study are included in the article/Supplementary Material, further inquiries can be directed to the corresponding author.
